# Designing and optimization of an electrochemical substitute for the MTT (3-(4,5-Dimethylthiazol-2-yl)-2,5-diphenyltetrazolium bromide) cell viability assay

**DOI:** 10.1038/s41598-019-51241-6

**Published:** 2019-10-18

**Authors:** Mohammad Mazloum-Ardakani, Behnaz Barazesh, Seyed Mohammad Moshtaghioun, Mohammad Hasan Sheikhha

**Affiliations:** 1 Department of Chemistry, Faculty of Science, Yazd University, Yazd, 89195-741 Iran; 2 Department of Biology, Faculty of Science, Yazd University, Yazd, Iran; 3 Research and Clinical Center for Infertility, Shahid Sadoughi University of Medical Sciences, Yazd, Iran

**Keywords:** Biochemical assays, Diagnostic markers

## Abstract

For the first time ever, this paper reports the development of an easily operated and cost-effective electrochemical assay to be used as an appropriate substitute for the MTT (3-(4,5-Dimethylthiazol-2-yl)-2,5-diphenyltetrazolium bromide) cell viability assay. The proposed assay is based on the electrochemical reaction of *Saccharomyces cerevisiae* (*S*. *cerevisiae*) with toxic materials, and it overcomes most of the limitations of MTT such as evaporation of volatile solvents, cytotoxic effects of MTT reagents, high cost, and sensitivity to light. The novel electrochemical assay can be used to detect diazinon in the range of 10^−6^ g mL^−1^ to 10^−2^ g mL^−1^ with the detection limit of 1.5 × 10^−7^ g mL^−1^.

## Introduction

In recent years, environmental pollution has been taken into substantial consideration due to its detrimental effects on the health of human beings and other living organisms. In this regard, industrial development has served as a source of various environmental problems mainly through plentiful toxic compounds released into nature^1^.

The 3-(4,5-Dimethylthiazol-2-yl)-2,5-diphenyltetrazolium bromide (MTT) assay is a dye compound used as a colorimetric assay to assess cell metabolic activities. It can also be used to measure the cytotoxic or cytostatic activities of potential medicinal agents and toxic materials. Once the MTT assay is used, the water-soluble yellow dye MTT is converted into an insoluble purple formazan by the action of mitochondrial reductase through oxidoreductase enzymes^2^.

Cytotoxicity can cause damage by decreasing the number of active mitochondria in the cells. If the mitochondrial activity is too low in a cell, it might stop working properly. Mitochondrial activity and cellular viability can be assessed using an MTT viability assay with which to measure cytotoxicity.

The MTT assay, which replaced the radioactive tritiated thymidine incorporation assay, is, indeed, the first widely accepted method of measuring cell viability. However, there are certain limitations^3^ with the use of this assay as follows:

- Volatile solvents evaporate.
- As reported, the color of acid-isopropanol solubilized formazan remains stable for a few hours at room temperature.
- Due to the cytotoxic effects of MTT reagents, adding these materials to estimate cell viability may damage the cells or even kill them during an experiment
- Since MTT reagents are sensitive to light, MTT assays have to be done in dark.
- MTT is a costly method.

To evade the above-mentioned limitations, this paper proposes a novel, easily operated, and cost-effective electrochemical assay to be used as an appropriate substitute for the MTT assay. This means that, the electrochemical assay can be used for measuring toxicity of chemicals through measuring cell viability.

As mentioned above, chemical contaminants can be spread through water and food and find their way to human body. These contaminants can effect human health and cause different kind of disease such as cancer.

Hence, it is essential to have an appropriate assay for measuring and monitoring chemical contaminants.

At current work, a yeast-based biosensor (YBB) is employed based on the reaction of *Saccharomyces cerevisiae* (winemaking and baking yeasts) with potential medicinal agents and toxic materials at the presence of an electrochemical mediator (Fe(III)).

According to the Rawson *et al*. work^4^, an electrochemical mediators (such as is Fe(III)) can be reduced to Fe(II) by the catabolic redox sites of eukaryotic cells (such as Saccharomyces cerevisiae).

To evaluate the YBB assay, the reaction of *Saccharomyces cerevisiae* (*S*. *cerevisiae*) and diazinon (DA) is investigated.

Diazinon^5,6^^,^, as an organophosphate insecticide, was widely used to control cockroaches, silverfish, ants, and fleas in residential areas. The use of this chemical in residential areas was banned by the law in the U.S. in 2004, but it is still permitted for agricultural usages. Food contamination by diazinon may pose dangers to humans. Diazinon poisoning is associated with several symptoms such as difficult breathing, lack of urine flow control, feeling of weakness, headache, and blue lips and fingernails. Also, through the generation of free radicals, diazinon causes oxidative damages, lipid peroxidation and DNA fragmentation.

## Results and Discussion

### Electrochemical behavior of YBB

**Figure 1:**
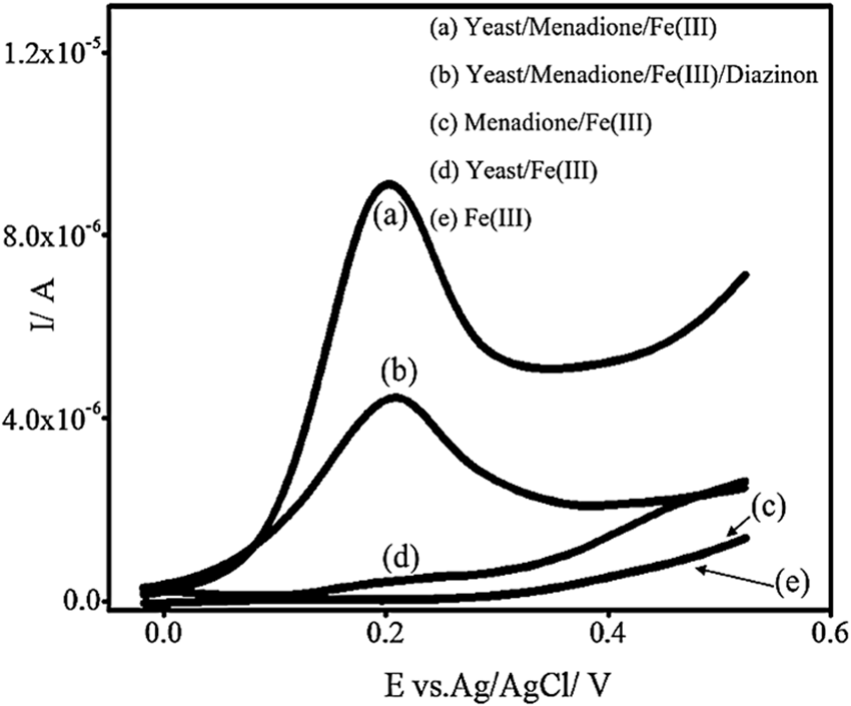
The electrochemical behavior of YBB assay with the LSV technique at room temperature and at pH 5.0 (the optimum pH for *S*. *cerevisiae* cells to grow and reproduce) by scanning from −20 to 550 mV at the scan rate of 100.0 mV/s.

As it can be seen in Fig. 1, at the beginning of the assay (the assay is according to the guidelines in the method section) there is only Fe(III) in the solution. According to the data, in the absence of yeast cells and MD, there is no reduction of Fe(III) to Fe(II) in the bulk solution; hence, no anodic current was observed (Fig. 1e) (As mentioned in the guideline: Eukaryotic cells (such as *Saccharomyces cerevisiae*) have most of the catabolic redox sites located in the cytoplasm and mitochondria. Electrochemical mediators are able to accept electrons from a eukaryotic cell through a number of pathways. An example of electrochemical mediators is Fe(III), which can be reduced to Fe(II) by the catabolic redox sites of eukaryotic cells. This reduction occurs parallel to the oxidation of NAD(P)H. However, hydrophilic mediators, such as Fe(III), cannot cross the cell membrane)^4,7^.

When Fe(III) was incubated with MD, no anodic current was observed, and it means that there is no reduction of Fe(III) to Fe(II) in the bulk solution, in the absence of yeast cells (Fig. 1c).

When Fe(III) was incubated with yeast cells, a relatively small proportion of Fe(III) was reduced to Fe(II), which resulted from trans-plasma membrane electron transport systems (tPMETs)^4,7^. It is to be noted that tPMETs only transfer a small number of cellular electrons to the periplasm. This results in a fairly small anodic current (4.7 × 10^−7^ A), which results from oxidation of Fe(II) to Fe(III) at the positive potential of 200.0 mV (Fig. 1d).

Fe(III) is hydrophilic and can only interact with redox sites embedded in the cell membrane and exposed to the periplasm. Hence, after the incubation of Fe(III) with yeast cells, glucose, and the lipophilic mediator (MD) within an optimum time, the anodic current increased to 9.2 × 10^−6^ A. The increase in the current arose from the oxidation of Fe(II) (Fig. 1a) (As mentioned in the guideline: In double-mediator systems, lipophilic mediators, such as menadione sodium bisulfite (MD) can cross the cell membrane of *Saccharomyces cerevisiae*, interact with intracellular redox centers, become reduced, and then move out of the cell to transfer electrons to a hydrophilic reporter mediator^4,7^, such as Fe(III), this means that the incubation of Fe(III) with *S*. *cerevisiae* and MD reduces Fe(III) to Fe(II) in a relatively large proportion^4^).

When Fe(III) was incubated with MD, yeast cells and DA, the peak current decreased. As mentioned before, DA caused a decrease in the number of the active mitochondria, which mainly resulted in a decrease of the anodic current (the anodic current decreased to 4.5 × 10^−6^ A) (Fig. 1b) (As mentioned in the guideline: toxic materials caused a decrease in the number of viable cells which mainly resulted in a decrease of the anodic current (compared to the anodic current of the blank sample).

## Optimization of Experimental Parameters

### The effect of initial pH

Extracellular pH has an important role in growth and the maintenance of the normal functions of yeast cells^8^. To investigate the effect of the pH on the yeast cells growth and the sensors operation, the cells were grown at different initial pH values and in the yeast growth media, as described above. Then, the cells were harvested and all the aforementioned guidelines were followed. According to the data (Fig. S1), pH 5.0 was the best pH for yeast cells to grow and reproduce.

A subject of focus in the present study is the effect of DA on the yeast cells. So, the best condition was chosen for growing the *S*. *cerevisiae* cells. In this respect, in aqueous solution and at room temperature, pH 5.0 was set as the optimum pH value for *S*. *cerevisiae* cells (pH 5.0 was the best pH for yeast cells to grow and reproduce, and after growing and reproducing of the yeast cells in the pH 5.0, the cells were harvested (collected by centrifuging at 3000.0 rpm, and washed with distilled water twice) and then added to a mixture of 5.0 mL of 1 mM ferric chloride solution) to grow and reproduce, (*Saccharomyces cerevisiae* is a kind of yeast which can grow at room temperature and that’s one of the advantages of using *Saccharomyces Cerevisiae* in the construction of the biosensor).

### The effect of MDs concentration

Due to its lipophilicity, MD is able to cross the cell membrane, enter the cell, and take electrons from a large number of redox molecules which exist inside the cell. MD, in a reduced form, gets back to the extracellular environment and transfers its electrons to Fe(III). This leads to the generation of Fe(II) which becomes oxidized at the electrode surface^4^. Various concentrations (10.0 μL, 30.0 μL, 50.0 μL, 100.0 μL and 200.0 μL of 100.0 mg/mL menadione sodium bisulfite) was tested and according to peak currents, the optimum concentration of MD for the solution (prepared on the basis of step 1 and 2 in the guidelines) was 100.0 μL of 100.0 mg/mL menadione sodium bisulfite. Also, as the results suggest, a higher concentration of MD would not increase the peak current, which means that 100.0 μL of 100.0 mg/mL menadione sodium bisulfite was enough to complete the assay in the mentioned concentration (Fig. S2).

### The effect of diazinon contact time on yeast cells

In this study, LSV was used to investigate the effect of DA contact time on yeast cells. To do this, for each time t, two parallel samples were prepared. One of them served as the blank sample and contained a mixture of *S*. *cerevisiae* (prepared on the basis of step 1 in the guidelines), then 5.0 mL of 1 mM ferric chloride (Fe(III)) and 50.0 μL of 100.0 mg/mL menadione sodium bisulfite, were added to the above-mentioned solution (on the basis of step 2 in the guidelines) and its signal measured by LSV was considered as I_1_.

The second sample contained ferric chloride Fe(III), MD, and *S*. *cerevisiae* in the same concentrations as before, but DA was added to them. This sample, too, was shaked (at 80 rpm) for the same time as the blank sample (i.e. t_1_), and its LSV signal was considered as I_2_. To investigate the effect of DA contact time, ΔI (I_1_−I_2_) was plotted versus the time t.

**Figure 2:**
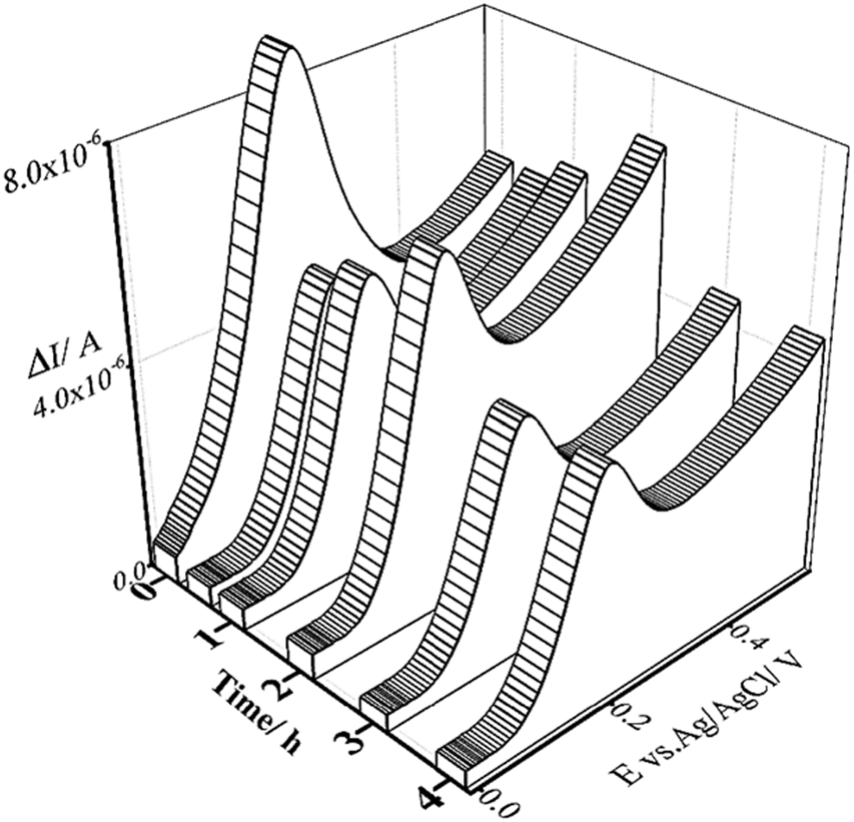
The effect of the contact time of yeast cells with diazinon using the LSV technique at room temperature and at pH 5.0 (the optimum pH for *S*. *cerevisiae* cells to grow and reproduce) by scanning from −20.0 to 550.0 mV at the scan rate of 100.0 mV/s.

As it can be seen in Fig. 2, at the time 0 h (0 h means that the there is still no toxic material (diazinon) in aqueous solution) an anodic current was observed which is due to the incubation of Fe(III) with yeast cells, glucose, and menadione.

After incubation of the yeast cells with DA for half an hour, the anodic peak current decreased. This was probably due to the influence of DA on the yeast cells, which caused the cellular metabolism to stop for a short while, leading to a decrement in the mitochondrial activity against toxic species.

As the experiment went on for about two hours, the anodic peak current increased, which was the result of the mitochondrial stress response that led to an increase in the mitochondrial activity (as the major intracellular source of reactive oxygen species) to limit the effect of the toxic species (i.e. diazinon). Reactive oxygen species (ROS) were generated by NAD(P)H oxidases; hence, the anodic peak current increased. An example of electrochemical mediators is Fe(II), which can be reduced to Fe(III) by the catabolic redox sites of eukaryotic cells^9^. This reduction occurs parallel to the oxidation of NAD(P)H.

After incubation of the yeast cells with DA for three hours, the anodic peak current decreased again and remained unchanged for the next hour. That was due to the excessive ROS production, which could lead to the oxidation of macromolecules. This process is implicated in mtDNA mutations, aging, and cell death^10^. According to the results, the optimum time of DA contact with yeast cells is three hours (According to the basic protocol of MTT Cell Proliferation Assay Instruction Guide^11^, the incubation of plate cells would take 6 to 24 hours, then, 10 μL of MTT reagent was added to the above mentioned medium and incubated for 2 to 4 hours (until purple precipitate is visible) and after that, the solution should remain in the dark for another 2 hours, it seems that the current works result is comparatively good, compare to the MTT result).

### The repeatability study

The investigation of the repeatability of the YBB assay was carried out with three assays which were made individually on three different days. In each assay, 0.5 g of tablet-form *S*. *cerevisiae* was added to three parallel yeast media each consisting of 1 g of glucose added to 100.0 mL of a nutrient broth solution. Then, the assays were treated according to the above-mentioned guidelines.

**Figure 3:**
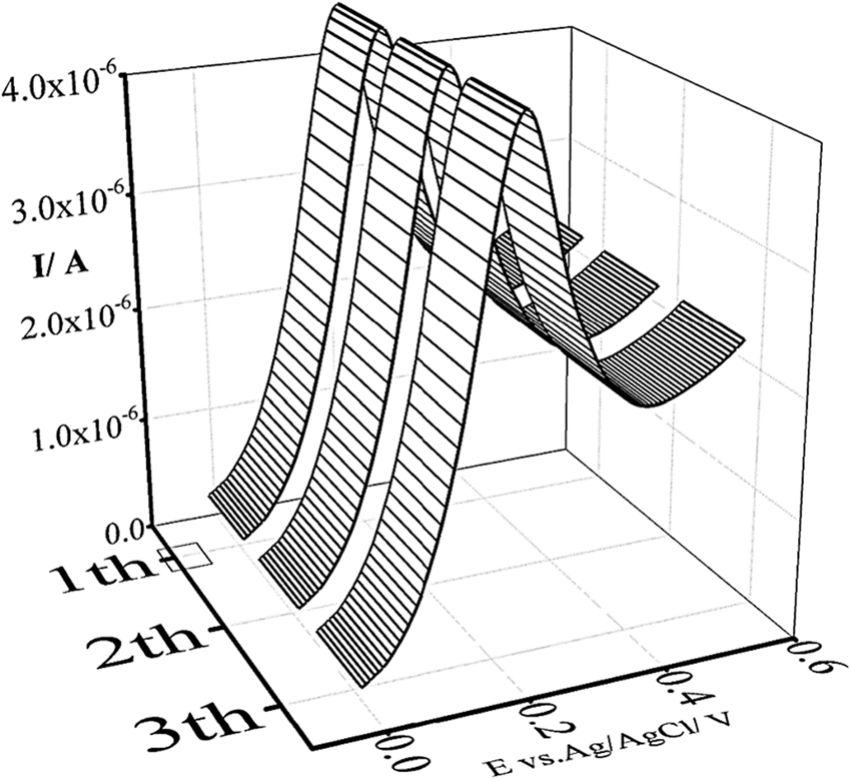
Study of repeatability using the LSV technique at room temperature and at pH 5.0 (the optimum pH for *S*. *cerevisiae* cells to grow and reproduce) by scanning from −20.0 to 550.0 mV at the scan rate of 100.0 mV/s.

The studies were done using the LSV analysis in 1 mM of a ferric chloride solution (pH = 5; pH 5.0 is the optimum pH for *S*. *cerevisiae* cells to grow and reproduce) on the surface of a GCE. The relative standard deviation of currents (A), providing an indication of variability, showed an acceptable result of 9 × 10 ^−3^ (Fig. 3).

### Sensitivity of YBB in the detection of diazione

**Figure 4:**
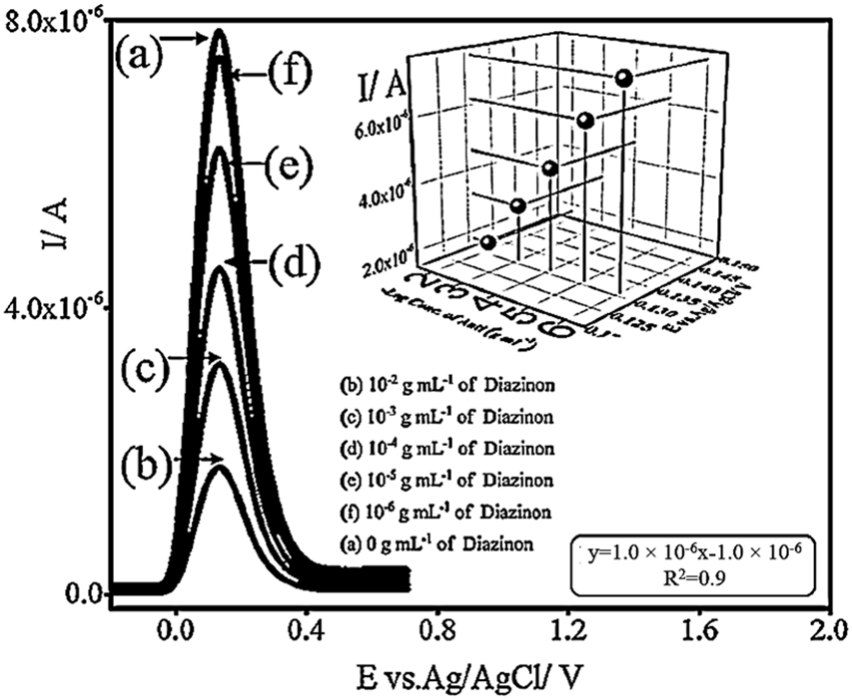
The sensitivity of YBB in the detection of diazinon with the SWV technique at room temperature and at pH 5.0 (the optimum pH for *S*. *cerevisiae* cells to grow and reproduce) by scanning from −20.0 to 710.0 mV.

The sensitivity of the YBB assay for the detection of diazinon was verified by using various concentrations of diazione. The results indicated that the SWV signals of the 1 mM ferric chloride solution (pH = 5.0; pH 5.0 is the optimum pH for *S*. *cerevisiae* cells to grow and reproduce) would decrease with an increase in the diazinon concentration. As it can be seen in Fig. 4, the peak current of 1 mM ferric chloride on YBB was linearly related to the negative logarithm of the concentrations of diazinon in the range of 10 ^−6^ g mL^−1^ to 10 ^−2^ g mL^−1^ with the detection limit of 1.5 × 10^−7^ g mL^−1^ for diazione (the linear regression equation fitted to the data is y = 1.0 × 10^−6^ × −1.0 × 10^−6^; R^2^ = 0.9).

## Conclusion

In this paper, a novel, easily operated, cost-effective electrochemical assay is proposed as an appropriate substitute for the MTT assay. It is, indeed, a yeast-based biosensor (YBB) developed on the electrochemical reaction of *S*. *cerevisiae* with toxic materials. The proposed assay can overcome most of the limitations of the MTT assay. To evaluate the YBB assay, the reaction of *S*. *cerevisiae* with DA was investigated. According to the results, the peak current of 1 mM of ferric chloride on the YBB is linearly related to the logarithm of the concentrations of diazinon in the range of 10^−6^ g mL^−1^ to 10^−2^ g mL^−1^ and the detection limits of 1.5 × 10^−7^ g mL^−1^ for diazione.

## Methods

### Chemical and reagents

Ferric chloride, sodium hydroxide (NaOH), glucose, nutrient broth, diazione, and Menadione sodium bisulfite (MD), as the chemicals used in the experiments, were of analytical grades. They were purchased from Sigma-Aldrich.

*S*. *cerevisiae* yeast was taken from the microbial collection of Yazd University, Yazd, Iran. All the solutions were prepared freshly with double distilled water.

The electrochemical measurements were performed using a μAutolab potentiostat/galvanostat, type (III). The experimental conditions were controlled with the General Purpose Electrochemical System (GPES) software (Kanaalweg 29/G, 3526 KM Utrecht, the Netherlands). To investigate the sensors, an Ag/AgCl/KCl (3.0 M) electrode, a platinum wire, and a glassy carbon electrode (GCE) were employed as the reference, auxiliary and working electrodes respectively.

The pH of the solutions was measured using a Metrohm 691 pH/ion Meter (Ionenstrasse 9101 Herisau Switzerland). A GFL1101 shaker (Schulze-Delitzsch-Strasse 4 30938 Burgwedel / Germany) was used to speed up the yeast growth by agitating the mixture.

### Pre-treatment of the working electrode

Before each experiment, the glassy carbon electrode was polished with slurry of 0.3 µm alumina. It was then washed with distilled water before each scan.

### Electrochemical measurements

In linear sweep voltammetry (LSV) measurements, the voltage is scanned from a lower potential limit to an upper potential limit.

In squarewave voltammetry (SWV) measurements, a combination of square wave potential and staircase potential applied to a working electrode.

At current work, LSV and SWV were applied to measure the anodic current which results from oxidation of Fe(II) to Fe(III).

It is notable that the SWV measurements have the advantage over LSV investigations. The advantage of SWV measurements in analytical investigations is due to the elimination of the capacitive/background current and that’s arose from sampling the current twice: The currents are investigated at the end of each pulse and the difference between these two currents (which were measured on two continuous pulses), is recorded as a net response^12^.

### Linear sweep voltammetry (LSV)

Linear sweep voltammograms were obtained in 1 mM of a ferric chloride solution (incubated with *S*. *cerevisiae* and MD) at room temperature and at pH 5.0 (pH 5.0 is the optimum pH for *S*. *cerevisiae* cells to grow and reproduce). It was done by scanning from −20.0 to 550.0 mV at the scan rate of 100.0 mV s^−1^ Hz vs. Ag/AgCl/KCl (3.0 M) electrode. The voltammograms showed a well-defined oxidation peak current on the GCE.

### Square wave voltammetry (SWV)

Square wave voltammograms were obtained in 1 mM of a ferric chloride solution (incubated with *S*. *cerevisiae* and MD), at room temperature and at pH 5.0 (pH 5.0 is the optimum pH for *S*. *cerevisiae* cells to grow and reproduce), and by scanning from −30.0 to 710.0 mV with a step potential of 20.0 mV, amplitude of 8.5 mV and frequency of 25.0 Hz vs. Ag/AgCl/KCl (3.0 M) electrode.

### Guidelines for the preparation of the YBB assay

The YBB assay is based on the electrochemical reaction of *S*. *cerevisiae* towards toxic materials.

**Figure 5:**
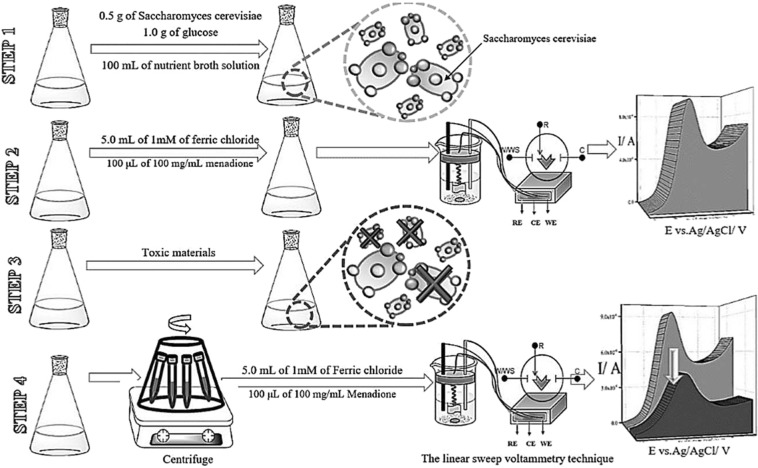
Guidelines for the preparation of the YBB assay.

This assay was prepared according to the guidelines below (see Fig. 5):

**Step 1:** 0.5 g of tablet-form *S*. *cerevisiae* and 1.0 g of glucose were added to 100.0 mL of a nutrient broth solution. The mixture was shaked (at 80 rpm) for 24 hours at the initial pH 5.0 (pH 5.0 is the optimum pH for *S*. *cerevisiae* cells to grow and reproduce) and at the temperature 28.0 °C.

**Step 2:** 10.0 mL of the above solution was added to a mixture of 5.0 mL of 1 mM ferric chloride (Fe(III)) and 100.0 μL of 100.0 mg/mL menadione sodium bisulfite.

Then the mixture was shaked (at 80 rpm) for an hour, at the temperature 28.0 °C. This one served as the blank sample, the blank sample was shaked for the optimum time and at the temperature 28.0 °C and its signal measured by LSV was considered as I_1_.

**Figure 6:**
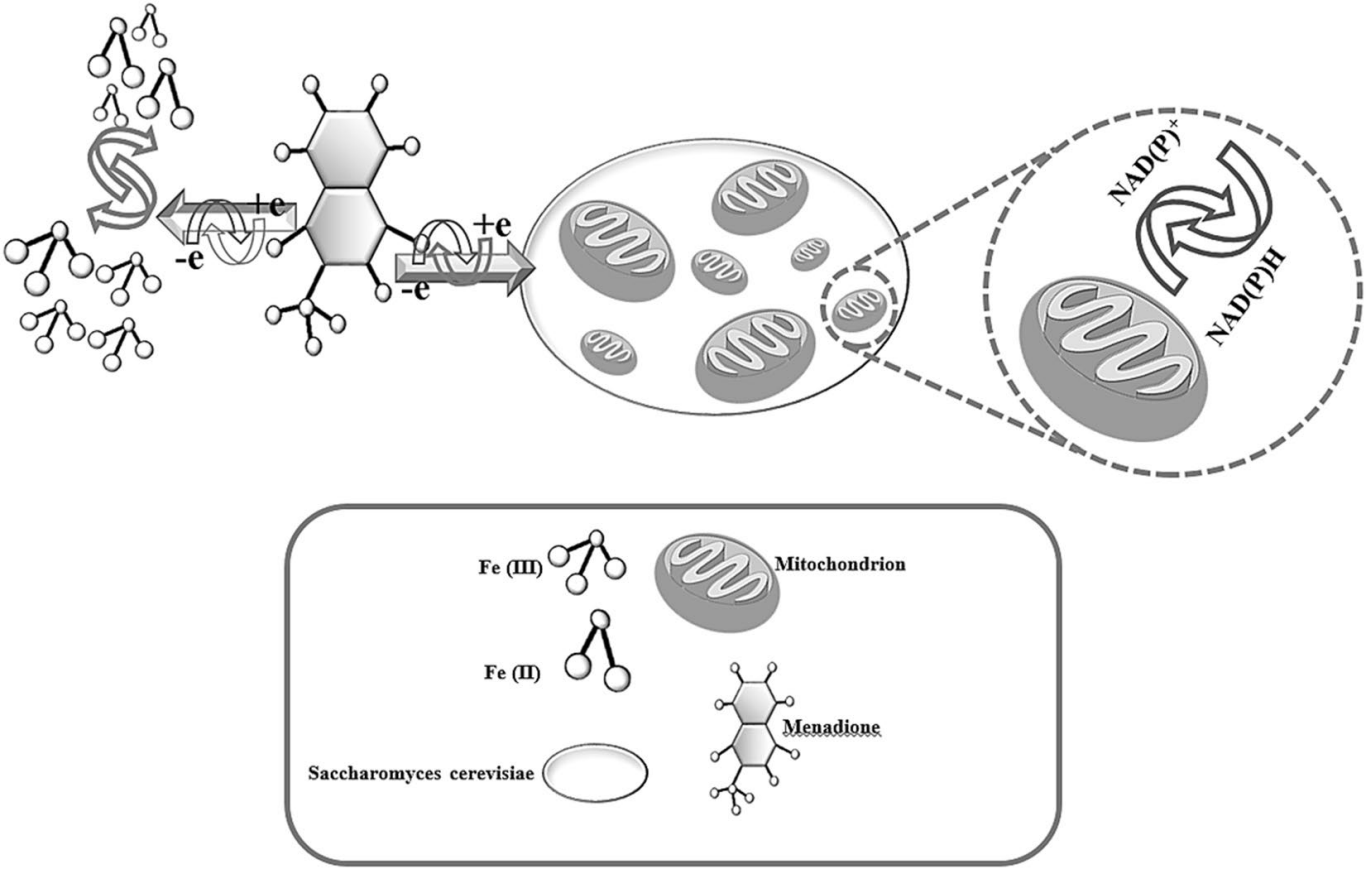
Schematic of a diagrammatic representation of the possible electrochemical mechanisms.

Note: Eukaryotic cells (such as *Saccharomyces cerevisiae*) have most of the catabolic redox sites located in the cytoplasm and mitochondria. Electrochemical mediators are able to accept electrons from a eukaryotic cell through a number of pathways. An example of electrochemical mediators is Fe(III), which can be reduced to Fe(II) by the catabolic redox sites of eukaryotic cells. This reduction occurs parallel to the oxidation of NAD(P)H. However, hydrophilic mediators, such as Fe(III), cannot cross the cell membrane. In this case, a double-mediator system which simultaneously has both lipophilic and hydrophilic mediators makes intracellular redox systems accessible. In double-mediator systems, lipophilic mediators, such as menadione sodium bisulfite (MD) can cross the cell membrane of *Saccharomyces cerevisiae*, interact with intracellular redox centers, become reduced, and then move out of the cell to transfer electrons to a hydrophilic reporter mediator^4,7^, such as Fe(III) (see Fig. 6).

According to the above-mentioned note, the LSV of Fe(III) only shows the cathodic current, which arises from the reduction of Fe(III) to Fe(II). The absence of an anodic current, as expected, indicates that there is no Fe(II) in the bulk solution.

In the presence of *S*. *cerevisiae* and MD, however, anodic voltammograms which arise from the oxidation of Fe(II) to Fe(III) do appear. This means that the incubation of Fe(III) with *S*. *cerevisiae* and MD reduces Fe(III) to Fe(II) in a relatively large proportion^4^.

**Step 3:** Certain amounts of toxic materials were added to 0.5 g of tablet-form *S*. *cerevisiae* and 1.0 g of glucose were added to 100.0 mL of a nutrient broth solution (prepared according to step 1). The mixture was shaked for an optimum time at the initial pH of 5.0 (pH 5.0 is the optimum pH for *S*. *cerevisiae* cells to grow and reproduce) at 28.0°C.

**Step 4:** The *S*. *cerevisiae* cells were collected by centrifuging at 3000.0 rpm, washed with distilled water twice, and then added to a mixture of 5.0 mL of 1 mM ferric chloride, Fe(III), and 50.0 μL of 100.0 mg/mL menadione sodium bisulfite. The solution was shaked for the optimum time and at the temperature 28.0°C and its signal measured by LSV.

Note: As expected, toxic materials caused a decrease in the number of viable cells which mainly resulted in a decrease of the anodic current (compared to the anodic current of the blank sample which was mentioned in step 2).

On the basis of the results, the assay proposed in this study can be claimed to be liable enough to substitute MTT. It is a simple electrochemical assay rather than a colorimetric one.

**Step 5:** The calibration curve was obtained through plotting the values of I_peak_ versus the negative logarithm of different concentrations of the toxic materials.

## Supplementary information

Supplementary
