# Supplementary material for: Designing and optimization of an electrochemical substitute for the MTT (3-(4,5-Dimethylthiazol-2-yl)-2,5-diphenyltetrazolium bromide) cell viability assay
Source: Sci Rep. 2019 Oct 18;9:14966. doi: 10.1038/s41598-019-51241-6 (PMC6802382; doi:10.1038/s41598-019-51241-6)
Supplement: Supplementary file 1 — Supplementary [file 41598_2019_51241_MOESM1_ESM.docx]

**Supporting information**

**Designing and optimization of an electrochemical substitute for the MTT (3-(4,5-Dimethylthiazol-2-yl)-2,5-diphenyltetrazolium bromide) cell viability assay**

Mohammad Mazloum-Ardakani^a*^, Behnaz Barazesh^a^, Seyed Mohammad Moshtaghioun^b^, Mohammad Hasan Sheikhha^c^

^a^ Department of Chemistry, Faculty of Science, Yazd University, Yazd, 89195-741, Iran

^b^ Department of Biology, Faculty of Science, Yazd University, Yazd, Iran

^c^ Research and Clinical Center for Infertility, Shahid Sadoughi University of Medical Sciences, Yazd, Iran

**Abstract**

For the first time ever, this paper reports the development of an easily operated and cost-effective electrochemical assay to be used as an appropriate substitute for the MTT (3-(4,5-Dimethylthiazol-2-yl)-2,5-diphenyltetrazolium bromide) cell viability assay. The proposed assay is based on the electrochemical reaction of *Saccharomyces cerevisiae* (*S*. *cerevisiae*) with toxic materials, and it overcomes most of the limitations of MMT such as evaporation of volatile solvents, cytotoxic effects of MTT reagents, high cost, and sensitivity to light. The novel electrochemical assay can be used to detect diazinon in the range of 10 ^-6^ g mL^-1^ to 10 ^-2^ g mL^-1^ with the detection limit of 1.5 × 10 ^-7^ g mL^-1^.

**Table of contents**

The effect of the initial pH …………………..……………………………..……………Figure S1

The effect of menadiones concentration …………………..…….……………………….Figure S2

**The effect of the initial pH**


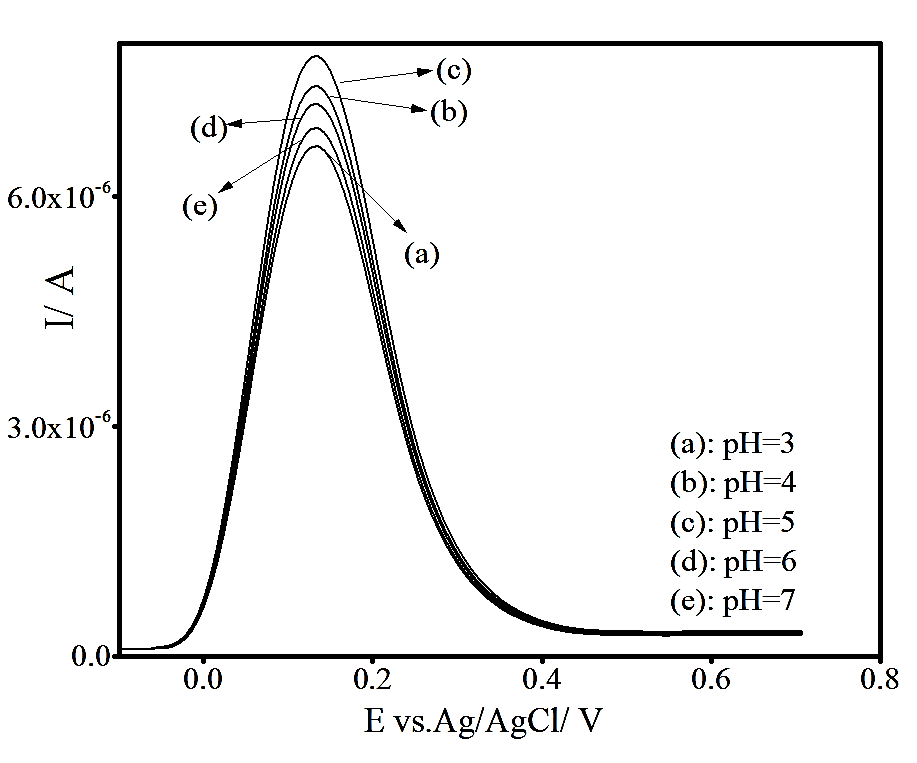


**Fig. S1.** The effect of the initial pH with the SWV technique at room temperature by scanning from -30.0 to 710.0 mV

**The effect of menadiones concentration**


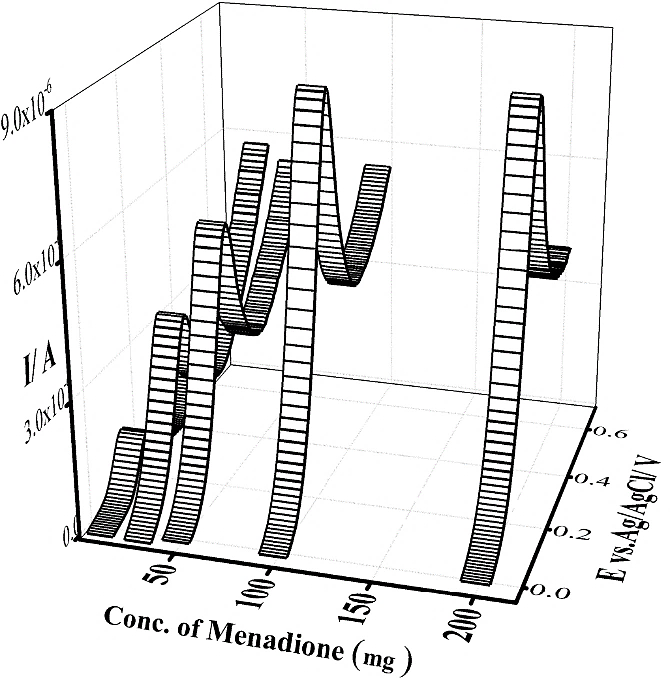


**Fig. S2.** The effect of MDs concentration with the LSV technique at room temperature and at pH 5.0 (the optimum pH for *S*. *cerevisiae* cells to grow and reproduce) by scanning from -20.0 to 550.0 mV at the scan rate of 100 mV/s
